# Editorial and Miscellaneous

**Published:** 1864-08

**Authors:** 


### EDITORIAL AND MISCELLANEOUS

CAMP NEAR PETERSBURG, VA., July 18, 1864.

*Messrs. Editors of Journal :—*

In the army, quinine is a fashionable treatment for all Idiopathic Fevers. Ask what is the rationale of this treatment, and the answer is, these fevers are caused by malarial poison, and quinine is therefore the sheet anchor. These fevers may be malarial, but there is a difference in the poison, or idiosyncrasy, of the patient, or his circumstances, which change the symptoms materially, from those cases in which I have found quinine to produce its good effects.

Three-fourths of the cases of fever in this army, that I have seen, are those in which there is no intermission. I do not say that the non intermission decides that there is no malarial influence, but it must be of that nature in which quinine is not useful.

The disease may commence with a slight chill or without one; the tongue is heavily coated with a whitish fur; generally there is severe headache, which, as usual, subsides in two or three days; not severe pain in the back; pulse from 100 to 130, etc. These cases are accompanied by considerable depression of the vital power. My experience in these cases leads me not to be favorably impressed by the quinine treatment. There is often a gastric irritability, with vomiting, which quinine increases; and it often increases the headache. I have seen it given in these fevers, in from two to five grain doses, continuing it for some time, but I never in one instance saw the good effect.

In many cases I could not see any difference in the progression of the case, whether quinine was given, or nothing used. It does not shorten the disease; often decreases the desire for food, which is already much below par. My remarks have been in reference to its use in large doses, in all stages of the disease, to break in upon the poison of the disease, and shorten it. But I will now say, that I imagine I have found it useful given in from one to two grain doses as a tonic, aided by stimulants, in those cases without gastric irritability, and after the skin has become moist.

Here, as in other lands, is found plenty of intermittent cases in which quinine does its work nobly. If quinine cures so certainly in these (intermittent) cases, it seems probable it might give more relief in the continued ones, if their poison was of the same nature. Tried practitioners of large experience, stating that the poison of these two classes of fevers is the same, leads the younger class to believe it true; but there is some difference in the poison, or idiosyncrasy of the patient, that causes a different effect of the same remedy.

In these fevers what is the treatment? It should be similar to our private typhoid fever, viz., give tonics to keep the vital powers that are held up by stimulants; and nourishing diet from the first day. I have found that the cooks of officers, who, of course, have a better bill of fare than the privates, when taken with this fever are not so much depressed, and convalesce sooner than they do, and what is still more, not so many proportionally have the disease.

The treatment must be similar to other cases where there is failure of vital power; as, articles of food that are nutritious; cleanliness; tonics; and, as a carminative and stimulant, fluid ext. zingiberis and Jamaica rum; and, above all, kind words and encouragement of recovery.

G. D. Winch, Ass't Surg., 36th Reg. Wis. Vol.

---

St. Charles, Minn., July 18, 1864.

*Drs. Miller and Ingals:—*

Permit me to add to history a case of Extra Uterine Pregnancy. Patient--Mary Kingsley; æt. 35; first pregnancy; having been married eight years; had enjoyed good health until time of pregnancy; since, she has complained of illness.

March 1st. Felt quickening distinctly.

June 18th. Taken ill, pains experienced in left side, above pubis. Confinement was expected, and Dr. J. H. Sudduth, of this place was called.

July 2d. I was called in counsel. This condition, with partial delirium, continued.

July 12th. Fever present, delirium increased.

July 16th. Pains resembling labor, continued two hours, followed by an hour of rest. Death supervened.

Dissection. The abdomen, which was greatly distended, was found to contain a large quantity of liquid. The uterus was empty, about twice the size of an unimpregnated uterus. The os sufficiently dilated to admit a finger. A fœtus of about eight pounds in weight was found in left fallopian tube, head near umbilicus.

The uterus was drawn upward and to the right, which made it difficult to reach the os in examination. No signs of life of fœtus had been observed for about two weeks previous to the death of the patient. H. H. Guthrie, M. D.

---

Messrs. Editors:—*Erratum.*—In the June number of this *Journal*, p. 267, (lines 8 and 9,) the sense of the last paragraph of the article over the initials J. A. A., is remarkably *obscured* by the typographical blunder in putting the word "observe" instead of "obscure," as it should have been printed. In this case, at least, the "obscure" makes the matter clearer than to "observe" it. Please relieve my idea from *obscurity* by substituting the "obscure."

Respectfully Yours, J. A. A.

---

The Surgeon of the Alabama.—David Herbert Llewellyn, who perished in the noble performance of his duty in the late action off Cherbourg, was the son of the Rev. David Llewellyn, perpetual curate of Euston Royal, Wilts. He was educated at Marlborough College, was an articled pupil of Dr. Hassall, of Richmond, and subsequently studied his profession at Charing-cross Hospital from 1856 to 1859. He was Silver

Medalist in Surgery and Chemistry. Of a generous and ardent disposition, he gained the esteem and regard of all his fellow-students. He was with the *Alabama* throughout the whole of her eventful career, and was much respected by all on board. We are enabled to give a copy of the last letter which we believe he ever wrote. It was addressed to Mr. Travers, the resident medical officer of Charing-cross Hospital, and is as follows:

"Cherbourg, June 14th, 1864.

"*Dear Travers*:—Here we are. I send this by a gentleman coming to London. An enemy is outside. If she only stays long enough, we go out and fight her. If I live, expect to see me in London shortly. If I die, give my best love to all who know me. If Monsieur A. de Caillet should call on you, please show him every attention.

"I remain, dear Travers, ever yours,

"D. H. LLEWELLYN."

How poor Llewellyn did his duty, as a man and a surgeon, may be judged by the following touching episode which was seen to occur during the late battle: The whaleboat and the dingy, the only two boats uninjured, were lowered, and the wounded men placed in them, Mr. Fulham being sent in charge of them to the *Kearsage*. When the boats were full, a man who was unwounded endeavored to enter one, but was held back by the surgeon of the ship—Mr. Llewellyn. "See," he said, "I want to save my life as much as you do; but let the wounded men be saved first." "Doctor," said the officer in the boat, "we can make room for you." "I will not peril the wounded men," was his reply. He remained behind, and sank with the ship—a loss much deplored by all the officers and men.—*Lancet.*

There is something in the organization of man that makes him love hero-worship, and it is natural that the unselfish devotion of the surgeon of the Alabama should excite our admiration, and it may be a subject of just pride to the medical profession, that the only worthy exhibition of character that we know, as connected with the Corsair's history, emanated from a mind that had been elevated by its study and practice. Yet it is no more than is shown by multitudes of physicians who confront danger and suffer death from disease contracted by attendance on those laboring under infectious pestilence, and conspicuous examples of equal self-sacrifice are not uncommon, even among uneducated men. Who has forgotten that when the Hornet sunk the Peacock, during the war of 1812, three American sailors went down in the Peacock while endeavoring to save their enemies who had surrendered. The history of the Alabama does not speak well for the character of any man who was a willing agent in making up this history. She was built, equipped and manned by a people, ostensibly our friends. She never entered the port of one of the seceded States, and there was nothing Confederate about her, except her commander and the flag under which she sailed. Until her last fatal day she sedulously avoided meeting a war vessel on the open sea with all the cunning instinct of a coward, and when in the proximity of an armed enemy she repeatedly stole out, under the cover of darkness, from the neutral ports she had entered to augment her crew or procure munitions, and went over the ocean, lighting it up with the helpless, unarmed sailing vessels of the merchant service; and when a long experience with the feeble had emboldened her commander to accept a conflict with a vessel of equal strength, and after Capt. Semmes had surrendered himself and crew as prisoners, as many of them as were able took a dishonorable advantage of Capt. Winslow's humane efforts to save their lives, to make their escape.

No vessel that ever sailed on the ocean has left a more disgraceful record than the Alabama, and that surgeon Llewellyn was a voluntary and cordial actor in the work, makes us doubt the propriety of attempting to transform him into a hero. We should rather point to his example to illustrate what we have sometimes heard alleged as an unusual proclivity of parson's sons to fall into disreputable lives.

---

Death from Chloroform.—A writer in the *Medical Times and Gazette*, states that out of fifty-one cases of death from chloroform, thirty-eight declared their danger by sudden stoppage of the pulse; twenty-five of these showed in addition as a chief sign, pallor of the countenance. In two deaths the symptoms have occurred thus: Sudden vomiting, instant cessation of the pulse, (food had been taken just before). In six cases, congestion of the face was the most marked symptom. In eight cases, cessation of breathing was the marked symptom. There is only one perfect stimulus to the failing heart, the stimulus of ærated blood. The means for producing this is by the excitation of artificial respiration, the tongue being drawn forward, and the introduction of nitrous oxide, either in its gaseous form, through the lungs, or condensed in water and introduced into the alimentary canal by the mouth or bowels.

---

The many friends of Dr. Powell will read the following, from the Vicksburg *Herald*, with pleasure:

McPherson Hospital.—No one visits this Hospital without being impressed with the consummate skill and business-like system with which it is managed. It is indeed a cheerful and quiet place for the afflicted. Dr. Powell, the gentlemanly surgeon in charge, is a man pre-eminently fitted for the position. Cleanliness, abundance of ventilation, and systematized management are the attractive features, and evidently enter largely into his ideas of good medical treatment. The wards are spacious, halls airy, with polished floors and high ceilings, and the rows of beds on either side, with their white, clean counterpanes, give to them an air of comfort and quiet rest which is equally attractive to those in health and strength as to the sick and disconsolate soldier.

We visited the hospital not long since, and found about one hundred and twenty sick men, and those in a state of rapid and permanent recovery. The good nursing and pure air will soon restore them to health and strength. It is gratifying to know that we have such a hospital with such a man at its head.

---

Dr. Alfred Stille has been elected Professor of the Practice of Medicine in the University of Pennsylvania, to fill the chair recently made vacant by the resignation of Dr. William Pepper; and Dr. B. Howard Rand has been elected to the chair of Chemistry, in the Jefferson Medical College, in the place of Prof. Franklin Bache, deceased.

---

*Philadelphia College of Pharmacy.*—Dr. Edward Parrish has been elected Professor of Materia Medica in this school, in place of the late Dr. Thomas.

---

*Tempting Offer.*—A man advertises for a competent person to undertake the sale of a new medicine, and adds that it will be profitable to the "undertaker."

---

The Paris Hospital Medical Society offers a prize of 1,000 francs for the best essay on the following subject: "Establish by means of positive facts the prophylaxis and curability of the form of Meningitis termed 'tubercular.'" To be written in French.

---

Dr. C. E. Brown-Sequard has returned to this country and taken up his residence in Boston.

---

The Confederate States Surgeon-General reports 2779 cases of disease, and 1396 deaths among the Union prisoners in Richmond, during the months of Jan'y, February and March last. Of the deaths, 708 were from chronic diarrhœa. This shows a ratio of mortality fearfully large, as compared with the rebel sick in our hands.

---

The Buffalo *Medical and Surgical Journal* reports two fatal cases of trichiniasis as occurring near that city. A portion of muscle from these persons, and of sausages, such as they had eaten some weeks previously, showed the trichina spiralis in great abundance.

---

*Constipation.*—Trousseau declares Belladonna to be the remedy, *par excellence*, for habitual constipation. It does not purge, nor produce loose stools, but only renders defecation easier, and sometimes in the dose of a quarter of a grain, the extract will produce several stools. As soon as the bowels become regular the dose of the medicine should be gradually diminished. Cases illustrative of the efficacy of this treatment are reported by Feissenger, who, however, made use of suppositories containing the ext. of belladonna; by Blache, also; and by Fleury.

---

In a late discussion at the Parisian Surgical Society, on amputations, M. Broca observed that statistics proved little or nothing in the matter. Amputations made in Paris and in the provinces were followed by very different results. In the provinces, amputation of the thigh generally succeeds, but in Parisian hospitals it is an operation of extreme danger, death being the rule and recovery from it the exception. Statistics, according to M. Broca, show that the mortality in Paris hospitals, after amputation of the thigh for injuries, is 100 per cent. Trélat makes it 83 in 100 cases.

---

*To Render the Taste of Medicine Palatable.*—It has been ascertained by M. Graw that the intensely bitter and nauseous taste of many drugs may be completely disguised by mixing them with chloroform. It is claimed that even the bitter taste of quinia and the peculiar odor of assafœtida can be thus destroyed.

---

*Camp Diarrhœa.*—This common and obstinate disease, so little amenable to treatment, has been found by Dr. Davis, of the 34th Iowa Vol., to readily yield to the following formula:

℞. Spir. Ætheris Nit., ℥ iij; Tinct. Opii, ℥ ij; Strychnia, gr. j. M. Give from thirty to forty drops four times a day. A majority of cases yield in 48 hours. So says the Doctor.

---

*Married.*—At the residence of the bride's father, in Royal Centre, Ind., by Rev. Wm. Griggsby, Israel B. Washbu rne, M. D., Surgeon 46th Ind. Vet. Vols., and Miss Mattie A., daughter of G. B. Moore.

